# Characteristics of a COVID-19 confirmed case series in primary care (COVID-19-PC project): a cross‐sectional study

**DOI:** 10.1186/s12875-021-01419-7

**Published:** 2021-04-08

**Authors:** Eloisa Rogero-Blanco, Vera González-García, Rodrigo Medina García, Pilar Muñoz-Molina, Santiago Machin-Hamalainen, Juan A. López-Rodríguez, Grupo COVID-AP, Francisco Abellán-López, Marta Barranco-Apoita, Carlos Bernaldo-de-Quirós, Manuel M. Bernaldo-Corral, Marta Bosom-Velasco, Carlos Casado-Álvaro, Pilar Casado-Sanz, Pilar Chaves-Sánchez, Paulino Cubero-González, Eva de-la-Torre-Buedo, Patricia Docavo-Muñiz, Raquel Fernández-Díaz, Teresa Ferrer-Valeiras, José D. Garcés-Ranz, Celeste García-Galeano, Jorge Gómez-Ciriano, Soledad Gómez-Criado, Marta Herranz-López, Josefina Hontanilla-Calatayud, Jorge Hurtado-Gallar, Pablo Jerez-Fernández, Juan A. López-Rodríguez, Santiago Machín-Hamalainen, Jacinto Macías-Rodríguez, Teresa Marín-Becerra, Raquel Mateo-Fernández, Rodrigo Medina-García, Paz Moldes-Rodríguez, Sara Morcillo-Cebolla, Purificación Pajares-Box, Lydia Palacios-Goncalves, Guilherme A. Preto-Berdeja, Asunción Prieto-Orzanco, Lara Quintana-Arencibia, Elosia Rogero-Blanco, Tomás Rossignoli-Fernández, María J. San-Telesforo-Navarro, Sara Sánchez-Barreiro, Laura Santos-Franco, Clara Vila-I-Torello, Teresa Ferrer-Valeiras, Ana Alejano-Rodríguez, Pedro Barbero-Sacristán, María Barranco-Camino, Isabel Calvo-García, Concepción Diaz-Calera, Yasmin Drak-Hernández, JuanCarlos Fuentes-Barona, Leticia Galtier-Gómez, Esperanza Gómez-Fernández, Vera González-García, Rocío Horcajada-Alocén, Isabel Hortelano-Galán, Pilar Muñoz-Molina, Belén Navarro-Carnero, Francisco C. Pérez Sánchez, Isabel Sáenz-García-Baquero, Vicente I. Torralba-Garrido, Francisco J. Zufia-Garcia, Andrea Valcarcel-Alonso

**Affiliations:** 1 Primary Health Care Centre General Ricardos, Calle General Ricardos 131, 28019 Madrid, Spain; 2 Health Services Research On Chronic Patients Network (REDISSEC), Madrid, Spain; 3 Primary Health Care Centre Los Rosales, Madrid, Spain; 4 Medical Specialties and Public Health Department, School of Health Sciences, University Rey Juan Carlos Alcorcón, Madrid, Spain; 5 Research Support Unit, Primary Care Management, Madrid, Spain; 6 Hospital Clínico San Carlos, Madrid, Spain

**Keywords:** COVID-19 [Supplementary Concept], Coronavirus Infections, Pneumonia, Viral, Primary Health Care

## Abstract

**Background:**

To estimate the prevalence of symptoms and signs related to a COVID-19 case series confirmed by polymerase chain reaction (PCR) for SARS-CoV-2. Risk factors and the associated use of health services will also be analysed.

**Methods:**

Observational, descriptive, retrospective case series study. The study was performed at two Primary Care Health Centres located in Madrid, Spain. The subjects studied were all PCR SARS-CoV-2 confirmed cases older than 18 years, diagnosed from the beginning of the community transmission (March 13) until April 15, 2020. We collected sociodemographic, clinical, health service utilization and clinical course variables during the following months. All data was gathered by their own attending physician, and electronic medical records were reviewed individually. Statistical analysis: A descriptive analysis was carried out and a Poisson regression model was adjusted to study associated factors to Health Services use.

**Results:**

Out of the 499 patients studied from two health centres, 55.1% were women and mean age was 58.2 (17.3). 25.1% were healthcare professionals. The most frequent symptoms recorded related to COVID-19 were cough (77.9%; CI 95% 46.5–93.4), fever (77.7%; CI95% 46.5–93.4) and dyspnoea (54.1%, CI95% 46.6–61.4). 60.7% were admitted to hospital. 64.5% first established contact with their primary care provider before going to the hospital, with a mean number of 11.4 Healthcare Providers Encounters with primary care during all the follow-up period.

The number of visit-encounters with primary care was associated with being male [IRR 1.072 (1.013, 1.134)], disease severity {from mild respiratory infection [IRR 1.404 (1.095, 1.801)], up to bilateral pneumonia [IRR 1.852 (1.437,2.386)]}, and the need of a work leave [IRR 1.326 (1.244, 1.413].

**Conclusion:**

Symptoms and risk factors in our case series are similar to those in other studies. There was a high number of patients with atypical unilateral or bilateral pneumonia. Care for COVID has required a high use of healthcare resources such as clinical encounters and work leaves.

## Background

As of December 31, 2019, the Chinese Authorities communicated to the WHO some pneumonia cases of unknown aetiology at Wuhan. A week later the Authorities confirmed it was caused by the new coronavirus called SARS-CoV-2 [1] (Severe Acute Respiratory Syndrome CoronaVirus 2).

In Spain, the first cases gradually began to appear since January 31 with a peak on March 20^th^ [2]. At the beginning of July 2020, the number of confirmed cases with a polymerase chain reaction (PCR) in Spain was over 251,000, and worldwide over 11 million cases [3]. In the Spanish National Seroprevalence Study (ENE-COVID) [4] a 5% prevalence was detected with rapid point of care antibody test and a 4.6% prevalence with the immunoassay test.

The symptoms associated with this viral infection have been called COVID-19 (Coronavirus Disease) and it includes respiratory symptoms such as a common cold, severe pneumonia, up to respiratory distress syndrome, septic shock and multiorgan failure. Initially, PCR test was not universally performed in those COVID-19 symptomatic patients.

From the clinical point of view, the most important published COVID-19 case series have been described in inpatients samples [5–7]. So far, there have been few published studies describing Primary Care (PC) case series [8]. The published studies reflect the disease semiology, as well as the main alterations of the complementary tests, risk factors, different treatments used and the disease evolution. With the RENAVE (National Epidemiological Surveillance Network) available data, the most frequent symptoms in PCR confirmed cases (*n* = 248,329) [9] were: fever or having had fever recently (72.9%), cough (69.0%), dyspnoea (47.6%) and diarrhoea (26.8%). But the real incidence of the clinical symptoms associated with the SARS-CoV-2 infection is unknown. In the report “COVID-19 Atención Primaria” [10] made by the Madrid Public Health System, in the Autonomous Community of Madrid 323,583 patients were attended in the PC Practices with COVID-19 confirmed or suspected. Out of those, only 52,902 (16.35%) were PCR confirmed; 53.8% of the PCR confirmed cases were diagnosed either of radiological or clinical pneumonia.

Since the change of epidemiological context and following the Spanish Ministry of Health instructions, mild COVID-19 cases should be diagnosed, followed up and epidemiologically surveilled at PC in coordination with the Public Health Care Services [11]. To perform those duties, PC had to reorganize its activities in order to lower the risk of contagion associated with crowds in closed facilities and prioritize emergency care and isolation of suspected cases [12]. To that effect, circuits inside the Health Centres had to be created to divide COVID-19 suspected cases assistance from other pathologies and, also, to prioritize telephone medical consults as well as home assistance.

In the Madrid region, 9.8% of the patients followed by PC since they had their first symptoms needed admission as inpatients. From the onset of symptoms, it took an average of 7.8 days to be admitted as inpatients [9]. Early detection of disease worsening of COVID-19 patients has been a priority for PC and it has been mainly done with follow-up phone consultations. So far there are no studies that analyse the socio demographic characteristics, symptoms, evolution and use of PC resources in Spain in this pandemic scenario.

The main objective of this study is to estimate the prevalence of symptoms and signs related to the COVID-19 infection confirmed by a PCR test for SARS-CoV-2, in patients older than 18 years in PC in the South Area of Madrid. Also, the risk factors and the use of healthcare resources associated with the COVID-19 infection will be analysed.

## Methods

Observational, descriptive, retrospective, followed-up case series study at two Primary Care Health Care Centres in the south area of Madrid.

Eligibility criteria were: a) older than 18 years b) having their attending Family Physician notified as PCR positive by the Official Public Health notification system c) being followed up (either by phone interviews or at the office) by their attending physician/nurse d) verbal informed consent to participate in the COVID-AP study. Exclusion criteria were: a) Institutionalized patients that were followed up by other sanitary professionals b) Those with mental disabilities or disorders whom their attending physicians judged couldn’t follow up the study requirements.

All cases that were notified in the electronical medical record (EMR) until the 15 of April 2020, by the Madrid Public Health Alert system, were analysed. Therefore, the sample was taken in a consecutive way as the cases were being notified from the referral hospitals and the Public Health Services to the attending physicians. The patients were followed up for 30 days after the positive PCR test was done. The data was gathered from May 16th until June 15th when the follow up period was over. 58 Family Physicians participated in the study and a total of 525 patients were confirmed and notified as PCR positive. Given a 11.5% of infected population estimation in the Madrid Autonomous Region, assuming the worst-case scenario for some symptoms (50%), and given a 95% confidence interval, the precision needed would be 4.27%.

### Variables

Main variables were symptoms related to the infection (fever, cough, odynophagia, dyspnoea, chills, vomits, diarrhoea, and anosmia/hyposmia) described in the patient's EMR throught all the follow up period disregarding the moment in which they appeared. Secondary variables were: 1. Sociodemographic: age, gender, nationality (Spanish/ Non-Spanish), being a healthcare professional 2. Patient’s comorbidities: high blood pressure (HBP), chronic obstructive pulmonary disease (COPD), asthma, diabetes, ischemic heart disease, cerebrovascular disease, chronic kidney disease, hepatic failure, immunosuppression, oncologic disease in the previous 5 years 3. Disease progression: first day with symptoms, first medical consultation date, positive COVID-19 test date, having been diagnosed of clinical pneumonia or radiologic pneumonia (unilateral or bilateral), 4. Use of resources: Primary Care contact before the PCR test, type of consult (phone, office), number of healthcare provider encounters (HPE) performed (before, during and after hospital care if needed), house calls made, need of hospital care at the Emergency Department, admitted as hospital inpatient, date of admission, date of discharge, place to be discharged, number of phone follow-ups after discharge, need of work leave and how many days of work leave were needed.

Family physicians collected the information from the patients’ EMR and their hospital discharge reports. Afterwards, they registered it anonymously into an online Data Collection Questionnaire (https://covidap.es/) with a random identification number. The data was gathered from May 16th until June 15th when the follow up period was over.

### Statistical analysis

The categorical variables were described as frequency and percentage; quantitative variables as average and standard deviation with a 95% confidence interval if they followed a normal distribution. In asymmetrical distributions, or not normal, the median and interquartile range were reported. The prevalence of the main symptoms detected were estimated with a 95% confidence interval. Bivariate analysis was done by groups (needing hospital care/not needing) using Chi square test for categorical variables and a Student’s T-test (or a Mann- Whitney U if the distribution was not normal) in the quantitative test. Multivariate Poisson regression model was adjusted to analyse associated factors being the number or HPE the dependant variable. All statistical analyses were performed using a standard software package (Stata, version 14.0).

All methods were carried out in accordance with relevant guidelines and regulations. Informed consent was obtained from all the participants.

#### Results

**Fig. 1:**
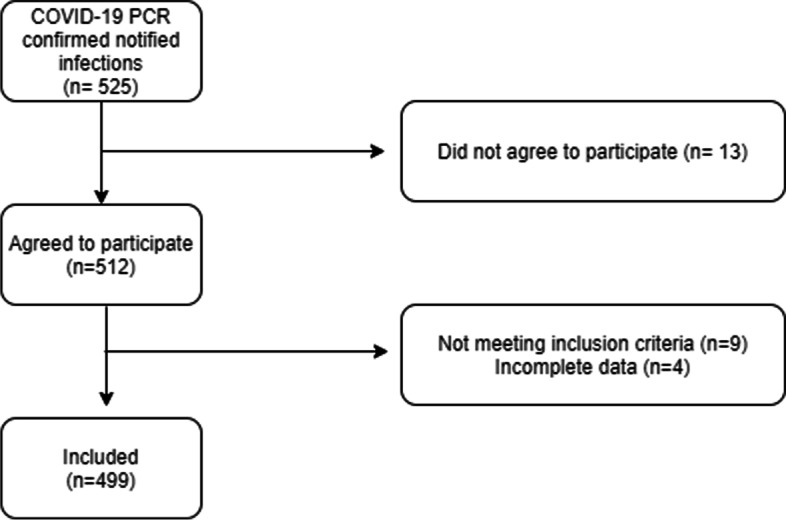
Patients included in the study

Out of the 525 patients that had a SARS- Cov-2 PCR confirmed infection, that was notified to their primary care physician, 499 were analysed (Fig. 1).

Out of these 499 patients: 275 (55.1%) were women, their average age was 58.2 years old (SD17.3) and 125 (25.1%) were healthcare workers. Most frequent risk factors were HBP (32.9%) and diabetes (17.5%). Table 1 summarizes the patients sociodemographic characteristics and risk factors.

**Table 1:** Sociodemographic characteristics, and risk factors of patients according to gender

	Total	Men	Women	*p* value
	499	224 (44.9%)	275 (55.1%)	
Age, mean (SD)	58.2 (17.3)	61.1 (17.2)	55.7 (17.1)	< 0.001 ^*¥*^
10 years age categories n(%)				0.003
18–25 years	15 (3.0%)	2 (0.9%)	13 (4.7%)	Ref. ^$^
26–35 years	38 (7.6%)	15 (6.7%)	23 (8.4%)	0.081
36–45 years	68 (13.6%)	28 (12.5%)	40 (14.5%)	0.058
46–55 years	117 (23.4%)	45 (20.1%)	72 (26.2%)	0.073
56–65 years	96 (19.2%)	38 (17.0%)	58 (21.1%)	0.066
66–75 years	69 (13.8%)	41 (18.3%)	28 (10.2%)	0.005
76–85 years	69 (13.8%)	40 (17.9%)	29 (10.5%)	0.006
> 85 years	27 (5.4%)	15 (6.7%)	12 (4.4%)	0.014
Country of origin				0.68
Spain	327 (65.5%)	147 (65.6%)	180 (65.5%)	
Others	145 (29.1%)	67 (29.9%)	78 (28.4%)	
NR/DK^*^	27 (5.4%)	10 (4.5%)	17 (6.2%)	
BMI, mean (SD)	28.5 (4.9)	28.7 (4.5)	28.4 (5.1)	0.53 ^*¥*^
Registered as smoker in the EMR	33 (7.8%)	22 (11.5%)	11 (4.8%)	0.011
Healthcare provider n(%)	125 (25.1%)	21 (9.4%)	104 (37.8%)	< 0.001
HBP	164 (32.9%)	91 (40.8%)	73 (26.5%)	< 0.001
Diabetes	87 (17.5%)	49 (22.0%)	38 (13.8%)	0.017
Asthma	36 (7.2%)	9 (4.0%)	27 (9.9%)	0.013
CAD	24 (4.8%)	21 (9.4%)	3 (1.1%)	< 0.001
Cerebrovascular disease	19 (3.8%)	16 (7.2%)	3 (1.1%)	< 0.001
COPD	24 (4.8%)	20 (9.0%)	4 (1.5%)	< 0.001
CKD	27 (5.4%)	16 (7.2%)	11 (4.0%)	0.12
Oncologic disease (in the last 5yrs)	40 (8.0%)	19 (8.5%)	21 (7.6%)	0.72

^*^NR/DK: Not response/doesn´t know; *EMR* Electronic Medical Record, *HBP* High Blood Pressure, *CAD* Coronary Artery Disease, *COPD* Chronic Obstructive Pulmonary Disease, *CKD* Chronic Kidney Disease, All analyses are Chi-Square test unless opposite specified; ¥ Student T test; $ Logistic univariate with reference category

Out of the 499 analysed patients, 488 (97.8%) had some symptom. Most frequent symptoms referred by the patients along the infection were: cough (78.0%; CI95% 46.5–93.4), fever (77.8%; CI95% 46.5–93.4) and dyspnoea (54.1%; CI95% 46.6–61.4). Anosmia (15.0%; CI95% 13.7–16.4) and ageusia /hypogeusia were less frequent (13.8%; CI95% 10.4–18.2). Among the main COVID-19 clinical diagnosis we found bilateral or severe pneumonia (45.5%) and mild respiratory infections (24.0%). 37 (7.4%) patients died, one of them at home. All symptoms analysed and main patients' diagnoses are shown in Table 2. 392 (78.6%) patients required hospital care, and 303 (60.7%) needed to be admitted as inpatients. Mean time from the beginning of the symptoms until the hospital admission as inpatient were 7.8 days (SD 5.2). Median time of hospital admission was 8 days (interquartile range from 5 to 14). 29 (9.3%) patients were admitted to the Critical Care Unit.

**Table 2:** Main symptoms referred during the disease and final diagnosis according to the need of being treated at the hospital or not

	Total (*n* = 499)	% (CI 95%)	Need of Hospital care^a^ (*n* = 392)	Didn´t require Hospital Care (*n* = 107)	*p* value
***Main Symptoms n(%)***					
Anosmia	75	15.03 (13.7–16.4)	46 (12.8)	29 (29.6)	< 0.001
Arthralgia/Myalgia	224	44.89 (5.7–91.6)	182 (50.3)	42 (42.0)	0.14
Asthenia	222	44.49 (27.9–62.4)	188 (51.8)	34 (34.3)	0.002
Diarrhoea	135	27.05 (11–52.8)	116 (32.0)	19 (19.2)	0.013
Dyspnoea	270	54.11 (46.6–61.4)	245 (65.0)	25 (25.5)	< 0.001
Chills	155	31.06 (9–67.3)	139 (38.7)	16 (16.7)	< 0.001
Fever	388	77.76 (46.5–93.4)	331 (88.0)	57 (56.4)	< 0.001
Hypogeusia	69	13.83 (10.4–18.2)	43 (12.0)	26 (26.5)	< 0.001
Nausea /Vomiting	70	14.03 (4.1–38.4)	62 (17.4)	8 (8.1)	0.023
Odynophagia	126	25.25 (14.8–39.7)	89 (24.9)	37 (37.0)	0.016
Skin reactions	18	3.61 (0.1–59.4)	13 (3.6)	5 (5.1)	0.51
Cough	389	77.96 (46.5–93.4)	314 (84.0)	75 (75.0)	0.038
***Final Diagnosis n(%)***					
Asymptomatic case	9 (1.8)		4 (1.0)	5 (4.7)	< 0.001
Mild respiratory disease	120 (24)		22 (5.8)	98 (91.6)	
Moderate respiratory disease	48 (9.6)		46 (12.0)	2 (1.9)	
Non severe or unilateral Pneumonia	62 (12.4)		61 (16.0)	1 (0.9)	
Bilateral Pneumonia	227 (45.5)		226 (59.2)	1 (0.9)	
Acute Respiratory Distress	23 (4.6)		23 (6.0)	0 (0.0)	
*Deceased n(%)*	37 (7.4)		36 (9.2)	1 (0.9)	< 0.001

^a^Need of hospital care: includes Emergency Department and hospital inpatients

Regarding the use of PC resources: 16 of April, 2020 was the date in which the highest number of first contacts was made with PC; 322 patients (64.5%) consulted PC prior to having a PCR for the first time. The average number of HPE in PC (with all the healthcare providers: physicians and nurses, and modalities: phone call or office visit, emergency or programmed visit) was 11.4 (6.8), during the next 30 days after the infection was confirmed; 53 patients (10.6%) needed home visits; 237 patients (75%) had instructions to be followed up in PC after being discharged; 225 patients (45.5%) needed a work leave certificate, which lasted an average of 31.8 days (SD15.4). In Table 3 a description of the PC resources used in the population according to needing hospital care or not.

**Table 3:** Use of Primary Care resources according to the need of being treated at the hospital or not

	Total (*n* = 499)	Need of Hospital Care (*n* = 392)	Didn´t require Hospital Care (*n* = 107)	*p* value
Mean appointments^a^ with PC x(SD)	11.4 (6.8)	11.6 (6.6)	0.21	0.21
Type of appointment the first-time n(%)				0.004
Phone Call	274 (59.8%)	202 (56.3%)	72 (72.7%)	
GP Surgery visit	184 (40.2%)	157 (43.7%)	27 (27.6%)	
Need of Housecall n(%)	53 (10.8%)	51 (13.2%)	2 (1.9%)	< 0.001
Asked to visit the surgery during follow-up n(%)	124 (25.6%)	104 (27.6%)	20 (18.7%)	0.063
Chest X-ray requested in PC n(%)	104 (21.1%)	94 (24.3%)	10 (9.3%)	< 0.001
Blood test requested in PC, n(%)	15 (3.0%)	11 (2.8%)	4 (3.7%)	0.75
Follow-up PCR requested in PC, n(%)	87 (17.8%)	48 (12.6%)	39 (36.4%)	< 0.001
Mean appointments after hospital discharge		6.1 (4.0)		
Work Leave needed, n(%)	225 (45.5%)	132 (34.0%)	93 (86.9%)	< 0.001
Mean days of Work Leave, x(SD)	31.8 (15.4)	36.7 (16.5)	25.9 (11.6)	< 0.001

*PC *Primary Care

^a^In the 30 days after diagnosis

Among the factors associated with the increase of HPE were: being male [IRR 1.072; CI95% 1.013, 1.134], mild respiratory infection [IRR 1.404; CI95% 1.095, 1.801], moderate respiratory infection [IRR 1.802; CI95% 1.372, 2.369], unilateral pneumonia [IRR 1.895; CI95% 1.467, 2.447], bilateral pneumonia [IRR 1.852; CI95% 1.437,2.386], respiratory distress syndrome [IRR 1.404; CI95% 1.055, 1.868)] and the need of a work leave [IRR 1.326; CI95% 1.244, 1.413]. Cerebrovascular disease was associated with less HCE [IRR 0.700; CI95% 0.590, 0.829)]. Univariate analysis and multivariate analysis can be found in Table 4.

**Table 4:** Associated factor related with the number of Healthcare Provider Encounters in PC

	**Univariate**		**Multivariate**	
	IRR	CI 95%	IRR*	CI 95%
Age	0.998^*^	[0.997,1.000]	1.001	[0.999,1.003]
Sex (Male)	1.042	[0.988,1.098]	1.072^*^	[1.013,1.134]
Body Mass Index	1.006	[1.000,1.012]		
Smoking history +	0.915	[0.821,1.019]		
High Blood Pressure +	0.963	[0.911,1.018]		
Chronic Obstructive Pulmonary Disease	0.794^***^	[0.694,0.909]		
Asthma	1.272^***^	[1.159,1.396]		
Diabetes	0.961	[0.896,1.030]		
Ischaemic Heart Disease	0.901	[0.792,1.026]		
Cerebrovascular Disease	0.654^***^	[0.555,0.772]	0.700^***^	[0.590,0.829]
Oncological disease	0.894^*^	[0.808,0.990]		
Chronic Kidney Disease	0.918	[0.813,1.037]		
Needing Hospital Care	1.088^*^	[1.020,1.161]		
Being Hospitalized as inpatient	0.895^**^	[0.832,0.964]	0.968	[0.893,1.049]
**Final Diagnosis**				
Asymptomatic	ref		Ref	
Mild Respiratory infection	1.490^**^	[1.163,1.910]	1.404^**^	[1.095,1.801]
Moderate Respiratory infection	1.592^***^	[1.215,2.086]	1.802^***^	[1.372,2.369]
Unilateral Pneumonia	1.802^***^	[1.400,2.319]	1.895^***^	[1.467,2.447]
Bilateral	1.646^***^	[1.287,2.105]	1.852^***^	[1.437,2.386]
Respiratory Distress Syndrome	1.246	[0.943,1.647]	1.404^*^	[1.055,1.868]
Work leave needed	1.254^***^	[1.190,1.321]	1.326^***^	[1.244,1.413]

Exponentiated coefficients; 95% confidence intervals in brackets; Multivariate Adjusted Poisson Regression Model

+ Not having means reference category

*Goodness of fit: AIC 3882.3, BIC 3928.4*

^*^p < 0.05, ^**^p < 0.01, ^***^p < 0.001

## Discussion

Out of a total of 499 patients studied from 2 different PC Health Centres at Madrid, the most prevalent symptoms associated to COVID19 infection were cough, fever and dyspnoea. HBP and diabetes were the most frequent risk factors. 25% of the patients were healthcare professionals. 60.7% required hospital admission as inpatients. 64.5% of patients had their first contact with a health care provider at PC. The average number of follow up contacts with PC was 11.4. The first contact with PC was mostly made by phone call. A work leave was needed by 45.5% patients with an average duration of 31.8 days.

Age and gender distribution in this case series is similar to previous studies [5–10, 13–15], although most of the other studies were made at hospital facilities. We have found only two other studies that used data from PC settings [8, 16, 17]. Most frequent symptoms were fever, cough and dyspnoea which coincide with most studies and official reports. In our study some symptoms, such as dyspnoea and cough, have higher prevalence. This fact could be explained due to the way the study was performed, in which while reviewing the clinical histories those symptoms were searched exhaustively from the very beginning to the end of the follow-up. Anosmia and ageusia were present in 15% and 13.8% of the patients of our study. These symptoms were first described in April 2020, with different prevalence according to the case series [18, 19].

Almost all of our patients were symptomatic and more than half of them had pneumonia (unilateral or bilateral). These results are above the ones given in the RENAVE COVID19[9] report and this difference could be explained by the way the sample was taken. In Spain at the beginning of the pandemic, only healthcare providers and the severe infections had a PCR test indicated and those tests were only performed at Hospital facilities. The same occurred to the hospitalization rates. The official guidelines to PCR test changed according to their availability and the community transmission status [20]. Since the data from the onset of the pandemic included only the PCR confirmed infections, some of the mild cases were not included in our study. Since May 2020, a PCR confirmation test was indicated for all clinical cases regardless of the severity of their symptoms or the health care provider (Hospital or Primary Care facilities)[11].

The use of PC resources has scarcely been studied at all. There are studies that highlight the PC attention as a main player in the pandemic and its fast adaptation to the new assistance model: prioritizing phone call consults and creating separated paths to treat patients with covid19 infection (confirmed or suspected) from non-COVID patients [21–27]. However, the burden it has meant to the healthcare providers and its costs have not been studied in detail. In our study, 64.5% of COVID19 patients contacted for the first time with PC, which supports the importance of PC in early detection. Also, the average number of 11.4 follow up contacts in the first months in those patients support the importance of Primary Care in containing of a pandemic.

The high percentage of patients (45.5%) needing a work leave increased the bureaucracy burden indeed and paperwork in the peak of the pandemic. Even the National Social Security Institute modified its procedures to facilitate patients’ confinement and communications with the companies the patients worked in. This increase in bureaucracy has also been described by other authors [28].

Among the factors associated with increasing the number of HPE we found disease severity and the need of a work leave. Even after adjusting with multivariate regression, they remained of statistical significance and some other important covariates that could be expected to influence that, such us respiratory diseases, dropped from the model. Also being male and having cerebrovascular disease were associated with the number of HPE and no explanation for that could be hypothesized. Further studies are needed and another HPE measurement at 6 or 12 months could be of help in that.

SaRS-CoV-2 has caused the first pandemic for a coronavirus, taking the health systems of different countries at the verge of collapse, and causing the closure of many institutions. The reorganization of the Health Systems to cope with this pandemic could lead us to an unequal distribution of the health resources, prioritizing medical treatment exclusively for COVID19 patients and unable to cope with other common acute and chronic conditions usually followed by PC.

### Strengths and weaknesses

On the one hand, one of the strongest points of our study was that the electronic medical record was reviewed exhaustively by their own family physician, who already knew the patient and frequently was in charge in most of the patient follow up. Also, the patients were followed up for 30 days after the positive PCR test was done. We do consider 30 days to be a longer follow up than other studies.

On the other hand, one limitation of this study was that since the data from the onset of the pandemic included only the PCR confirmed infections, some of the mild cases were not included in our study.

Other shortcoming could be that in the follow up we didn’t register when each new symptom appeared, therefore we can’t correlate the symptoms with the disease evolution.

## Conclusion

Symptoms and risk factors found in our case series in the South area of Madrid are similar to previous studies. The rate of unilateral or bilateral pneumonia in the confirmed cases was high. COVID-19 healthcare attention required the use of an elevated number of socio-sanitary resources. The health care system has been posed to its limits due to phone/office emergency and follow up consults, as well as time spent by physicians with work leaves, and other paperwork for COVID-19 patients, above the average Primary Care practices. Later, broader cost studies will be needed to quantify the impact in the health system.

## Data Availability

The datasets used and/or analysed during the current study are available from the corresponding author on reasonable request. Regarding data exchange, the Ethics Committee approved this research without considering the option of data sharing. The data contains sensitive clinical information about the patient, so there are ethical and legal restrictions to sharing the data set. The data are part of the COVID-AP study and can be requested by contacting the Ethics Committee at the email address ceic.hcsc@salud.madrid.org; or you can also contact the Primary Care Management of Madrid through the Technical Direction of Teaching and Research at the email address dtdei@salud.madrid.org. The COVID-AP Group may establish future collaborations with other groups based on the same data. The main researchers of the project will be contacted (Juan A López-Rodríguez at juanantonio.lopez@salud.madrid.org and info@covidap.es). However, each new project based on these data must be previously submitted to CEIC for approval.

## Abbreviations

PCR: Polymerase Chain Reaction
COVID: Coronavirus Disease
PC: Primary Care
EMR: Electronical Medical Record
HPE: Healthcare Providers Encounters
HBP: High Blood Pressure
COPD: Chronic Obstructive Pulmonary Disease
