# Correction to: Characteristics of a COVID-19 confirmed case series in primary care (COVID-19-PC project): a cross-sectional study

**DOI:** 10.1186/s12875-021-01458-0

**Published:** 2021-05-26

**Authors:** Eloisa Rogero-Blanco, Vera González-García, Rodrigo Medina García, Pilar Muñoz-Molina, Santiago Machin-Hamalainen, Juan A. López-Rodríguez, Grupo COVID-AP

**Affiliations:** 1 Primary Health Care Centre General Ricardos, Calle General Ricardos 131, 28019 Madrid, Spain; 2 Health Services Research On Chronic Patients Network (REDISSEC), Madrid, Spain; 3 Primary Health Care Centre Los Rosales, Madrid, Spain; 4 Medical Specialties and Public Health Department, School of Health Sciences, University Rey Juan Carlos Alcorcón, Madrid, Spain; 5 Research Support Unit, Primary Care Management, Madrid, Spain; 6 Hospital Clínico San Carlos, Madrid, Spain

**Correction to: BMC Fam Pract 22:66, (2021)**

10.1186/s12875-021-01419-7

In the original publication of this article, there was an error in the way the authors in the Grupo COVID-AP are mentioned. The names and surnames of the authors are not separated correctly by commas which makes it very difficult to find them when searching in Pubmed.

Collaborators

Grupo COVID-AP: Abellán López, Francisco Ramón, Barranco Apoita, Marta Bernardo de Quirós, Carlos Bernardo Corral, Manuel María, Bosom Velasco, Marta Casado Álvaro, Carlos Casado Sanz, M Pilar, Chaves Sánchez, Pilar Cubero González, Paulino De la Torre Buedo, Eva Docavo Muñiz, Patricia Fernández Díaz, Raquel Ferrer Valeiras, Teresa Garcés Ranz, José Damián, García Galeano, María Celeste, Gómez Ciriano, Jorge Ignacio, Gómez Criado, María Soledad, Herranz López, Marta Hontanilla Calatayud, Josefina Hurtado Gallar, Jorge Jerez Fernández, Pablo López Rodríguez, Juan Antonio, Machín Hamalainen, Santiago Macías Rodríguez, Jacinto Marín Becerra, María Teresa, Mateo Fernández, Raquel Medina García, Rodrigo Moldes Rodríguez, Mari Paz, Morcillo Cebolla, Sara Pajares Box, Purificación Palacios Goncalves, Lydia Preto Berdeja, Guilherme Artur, Prieto Orzando, Asunción Quintana Araencibia, Lara Rogero Blanco, María Eloísa, Rossignoli Fernández, Tomas San Telesforo Navarro, María José, Sánchez Barreiro, Sara Santos Franco, Laura Vila I Torello, Clara Ferrer Valeiras, Teresa Alejano Rodríguez, Ana Isabel, Barbero Sacristán, Pedro Barranco Camino, María Calvo García, Isabel Diaz Calera, M Concepción, Drak Hernández, Yasmin Fuentes Barona, Juan Carlos, Galtier Gómez, Leticia Gómez Fernández, María Esperanza, González García, Vera Horcajada Alocén, Rocío Hortelano Galán, María Isabel, Muñoz Molina, M Pilar, Navarro Carnero, Belén Pérez Sánchez, Francisco Carlos, Sáenz García Baquero, Isabel Torralba Garrido, Vicente Iván, Zufia García, Francisco Javier, Andrea Valcárcel Alonso.

We would like to change the way the authors in the Grupo COVID-AP are mentioned. The correct way should be:

Francisco Abellán-López, Marta Barranco-Apoita, Carlos Bernaldo-de-Quirós, Manuel M Bernaldo-Corral, Marta Bosom-Velasco, Carlos Casado-Álvaro, Pilar Casado-Sanz, Pilar Chaves-Sánchez, Paulino Cubero-González, Eva de-la-Torre-Buedo, Patricia Docavo-Muñiz, Raquel Fernández-Díaz, Teresa Ferrer-Valeiras, José D Garcés-Ranz, Celeste García-Galeano, Jorge Gómez-Ciriano, Soledad Gómez-Criado, Marta Herranz-López, Josefina Hontanilla-Calatayud, Jorge Hurtado-Gallar, Pablo Jerez-Fernández, Juan A López-Rodríguez, Santiago Machín-Hamalainen, Jacinto Macías-Rodríguez, Teresa Marín-Becerra, Raquel Mateo-Fernández, Rodrigo Medina-García, Paz Moldes-Rodríguez, Sara Morcillo-Cebolla, Purificación Pajares-Box, Lydia Palacios-Goncalves, Guilherme A Preto-Berdeja, Asunción Prieto-Orzanco, Lara Quintana-Arencibia, Elosia Rogero-Blanco, Tomás Rossignoli-Fernández, María J San-Telesforo-Navarro, Sara Sánchez-Barreiro, Laura Santos-Franco, Clara Vila-I-Torello, Teresa Ferrer-Valeiras, Ana Alejano-Rodríguez, Pedro Barbero-Sacristán, María Barranco-Camino, Isabel Calvo-García, Concepción Diaz-Calera, Yasmin Drak-Hernández, JuanCarlos Fuentes-Barona, Leticia Galtier-Gómez, Esperanza Gómez-Fernández, Vera González-García, Rocío Horcajada-Alocén, Isabel Hortelano-Galán, Pilar Muñoz-Molina, Belén Navarro-Carnero, Francisco C Pérez Sánchez, Isabel Sáenz-García-Baquero, Vicente I Torralba-Garrido, Francisco J Zufia-Garcia, Andrea Valcarcel-Alonso.

**Acknowledgements**

Grupo COVID-AP

**Primary Health Care Centre General Ricardos:** Francisco Abellán-López, Marta Barranco-Apoita, Carlos Bernaldo-de-Quirós, Manuel M Bernaldo-Corral, Marta Bosom-Velasco, Carlos Casado-Álvaro, Pilar Casado-Sanz, Pilar Chaves-Sánchez, Paulino Cubero-González, Eva de-la-Torre-Buedo, Patricia Docavo-Muñiz, Raquel Fernández-Díaz, Teresa Ferrer-Valeiras, José D Garcés-Ranz, Celeste García-Galeano, Jorge Gómez-Ciriano, Soledad Gómez-Criado, Marta Herranz-López, Josefina Hontanilla-Calatayud, Jorge Hurtado-Gallar, Pablo Jerez-Fernández, Juan A López-Rodríguez, Santiago Machín-Hamalainen, Jacinto Macías-Rodríguez, Teresa Marín-Becerra, Raquel Mateo-Fernández, Rodrigo Medina-García, Paz Moldes-Rodríguez, Sara Morcillo-Cebolla, Purificación Pajares-Box, Lydia Palacios-Goncalves, Guilherme A Preto-Berdeja, Asunción Prieto-Orzanco, Lara Quintana-Arencibia, Elosia Rogero-Blanco, Tomás Rossignoli-Fernández, María J San-Telesforo-Navarro, Sara Sánchez-Barreiro, Laura Santos-Franco, Clara Vila-I-Torello, Teresa Ferrer-Valeiras.

**Primary Health Care Centre Los Rosales:** Ana Alejano-Rodríguez, Pedro Barbero-Sacristán, María Barranco-Camino, Isabel Calvo-García, Concepción Diaz-Calera, Yasmin Drak-Hernández, JuanCarlos Fuentes-Barona, Leticia Galtier-Gómez, Esperanza Gómez-Fernández, Vera González-García, Rocío Horcajada-Alocén, Isabel Hortelano-Galán, Pilar Muñoz-Molina, Belén Navarro-Carnero, Francisco C Pérez Sánchez, Isabel Sáenz-García-Baquero, Vicente I Torralba-Garrido, Francisco J Zufia-Garcia.

**Hospital Clínico San Carlos:** Andrea Valcarcel-Alonso.

